# Accuracy and Efficacy of a 3D Dynamic Navigation System for Endodontic Retreatment: A Systematic Review and Meta-Analysis

**DOI:** 10.7759/cureus.93753

**Published:** 2025-10-03

**Authors:** Swapna Sannapureddy, Keerthana Laddagiri, Kiranmayi Govula, Niharika Mungara, Lavanya Anumula, Suneel Kumar Chinni

**Affiliations:** 1 Conservative Dentistry and Endodontics, Narayana Dental College and Hospital, Nellore, IND

**Keywords:** 3d guided endodontics, dynamic navigation system, endodontic retreatment file retrieval, post-removal, post-space preparation, real-time tracking technology, systematic review, systematic review and meta-analysis

## Abstract

The objective of this systematic review was to evaluate the accuracy of the dynamic navigation system (DNS) versus the freehand (FH) technique in nonsurgical endodontic retreatment, focusing on tooth structure loss, linear and angular deviations, iatrogenic errors, procedural time, and mishaps. Following the Preferred Reporting Items for Systematic reviews and Meta-Analyses (PRISMA) guidelines, an extensive literature search was conducted across several major databases, including PubMed, Scopus, the Cochrane Database of Systematic Reviews, and Web of Science, covering all available records from each database’s inception through August 2025. In addition, a search of the open gray literature was conducted, and relevant studies were identified through hand searching, yielding a total of 2,154 articles. After screening, 10 studies were included that compared DNS and FH in nonsurgical endodontic retreatment procedures, such as post space preparation and post removal. Among these, two studies focused on post space preparation, five on post removal, one on second mesiobuccal canal location, one on file retrieval, and one was a case report. The Quality Assessment Tool for In Vitro Studies (QUIN tool) was used to evaluate bias, indicating a moderate overall risk. Meta-analysis was performed on five studies: two on post space preparation and three on post removal. The DNS significantly reduced procedural time compared to the FH technique in both applications (p < 0.00001), although heterogeneity was high (I² = 95% and 59%, respectively). Compared to FH, DNS demonstrated better accuracy with less angular deviation (p < 0.0001 and p = 0.83; I² = 94% and 0%), coronal deviation (p = 0.06 and p = 0.12; I² = 72% and 59%), and apical deviation (p < 0.02 and p = 0.55; I² = 81% and 0%).

## Introduction and background

Guided endodontics is an advanced technique that utilizes custom-made 3D-printed splints or templates designed to assist treatment with either a static or dynamic guidance system [1,2]. The computer-guided dynamic navigation system (DNS) was initially developed to ensure precise implant placement [3,4]. Compared to static systems, DNS offers advantages in safety, comfort, accuracy, predictability, and procedural efficiency [5]. In DNS, the computer provides real-time feedback to the clinician regarding the drill path planned during treatment [6]. Multiple cameras and motion-tracking devices are connected to the patient and the dental handpiece, while specialized software continuously monitors deviations between the intended and actual drill trajectory using CBCT images of the teeth [1,7]. This allows intraoperative adjustment and reorientation of the drill, making DNS particularly beneficial for several endodontic procedures, including minimally invasive access cavity preparations [8], negotiating calcified canals [9], managing obliterated root canals [10], performing periradicular surgeries [11], and retreatment [12-14].

According to the American Association of Endodontists, nonsurgical endodontic retreatment is a complex procedure that involves the removal of previous obturating materials, retrieval of separated files, and removal of posts [15]. Challenges associated with retreatment protocols can be addressed through the combination of improved accuracy and reduced procedural time offered by advances in guided endodontics, such as DNS [16]. The literature must be systematically reviewed and critically evaluated to enhance understanding of DNS applications and their impact on retreatment outcomes. This systematic review and meta-analysis aims to assess the precision of DNS in nonsurgical endodontic retreatment compared with the conventional freehand (FH) approach. The evaluation focuses on parameters such as tooth structure preservation, drilling path efficiency, quantified by linear and angular deviations, iatrogenic complications, procedural duration, and operational mishaps.

## Review

Methodology

Protocol Registration

This systematic review, focusing on dynamic navigation, was conducted following the Preferred Reporting Items for Systematic reviews and Meta-Analyses (PRISMA) framework [17]. The review protocol was officially registered in the PROSPERO database under the registration number CRD42024541816. The research question was structured using the PICO format: Does dynamic navigation represent an accurate and efficient treatment modality in endodontic retreatment compared to conventional FH endodontic retreatment? The population included extracted human teeth, 3D-printed teeth, or individuals requiring endodontic retreatment. The intervention comprised procedures such as nonsurgical endodontic retreatment, post space preparation, post removal, and retrieval of separated endodontic files using 3D DNSs. The comparison involved performing the same procedures using the FH technique. The outcomes assessed were the accuracy of nonsurgical endodontic retreatment and/or tooth structure loss, as well as the efficiency of the drilling path measured by linear deviation (mm), angular deviation (degrees), and procedural time, with procedural mishaps as an additional outcome. The review included case reports, observational studies, in vitro studies, or ex vivo studies.

Search Strategy

An extensive literature search for this systematic review was conducted independently by two trained reviewers (SS and NM) across multiple electronic databases, including PubMed Central, Scopus, the Cochrane Database for Systematic Reviews, and Web of Science, covering all records from the inception of each database through August 2025. In addition, grey literature was searched through clinical trial registries, and four peer-reviewed journals, such as the International Endodontic Journal, Journal of Endodontics, Australian Endodontic Journal, and European Endodontic Journal, were hand-searched to identify relevant studies. The search strategy employed Medical Subject Headings (MeSH) and keywords in various combinations, including (retreatment OR root canal retreatment OR endodontic retreatment OR endodontic post removal OR endodontic file retrieval OR separated file removal) AND (dynamic navigation OR computer-aided technology OR computer-aided navigation OR computer-assisted treatment OR image-guided treatment OR real-time tracking).

Study Selection

Two researchers (SS and NM) independently screened the titles and abstracts of the retrieved records and removed duplicates. During the initial screening, narrative reviews, expert opinions, and guidelines were excluded. Studies were included if they were case reports, in vitro studies, or observational studies that used dynamic navigation for endodontic retreatment. Studies were excluded if they lacked comparison groups, used static navigation, or applied DNS to procedures other than retreatment. Discrepancies between reviewers regarding study inclusion were resolved through consensus with a third expert (CS).

Data Extraction

A customized data extraction form was developed by two reviewers (SS and NM) and completed for each included study. The form collected information on author, year, journal, study design, sample size, groups, dynamic software used, operator experience, outcomes assessed, and conclusions. Any discrepancies in the extracted data were resolved through consensus with a third reviewer (KG).

Quality Assessment

The quality of included studies was assessed independently by two reviewers (SS and KG) using the Quality Assessment Tool for In Vitro Studies (QUIN tool) [18]. This tool evaluates 12 criteria: clearly stated aims/objectives, sample size calculation, sampling technique, details of the comparison group, methodology, operator details, randomization, method of outcome measurement, outcome assessor details, blinding, statistical analysis, and presentation of results. Each criterion was scored as adequately specified (2), not adequately specified (1), not specified (0), or not applicable (NA). The overall risk of bias for each study was calculated using the formula:

$\text{Risk score (\%)} = \frac{\text{Score}}{2 \times \text{Number of applicable criteria}} \times 100$

Based on the scores obtained, studies were categorized as low risk (>70%), medium risk (50-70%), or high risk (<50%). Studies classified as high risk were excluded. Any disagreements between the two reviewers were resolved by consultation with a third reviewer (CS).

Results

Study Selection

**Figure 1:**
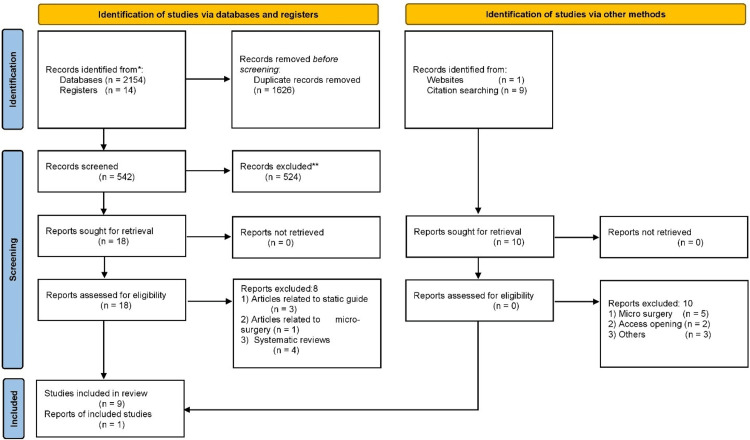
PRISMA flowchart PRISMA, Preferred Reporting Items for Systematic reviews and Meta-Analyses

A total of 2,154 articles were retrieved from the databases, 14 from registries, nine from hand-searching citations, and one from other websites. After removing duplicates, 542 articles were screened, and 18 were selected for full-text retrieval. Of these 18 articles obtained from databases, eight were excluded: three were related to static guides, one was related to microsurgery, and four were systematic reviews. All 10 articles obtained from other sources, such as citation searches, were excluded as follows: five were related to microsurgery, two were related to general endodontic treatment, and three were excluded for other reasons. The literature search revealed that no clinical trials had been published on the application of DNS in retreatment. Ultimately, a total of 10 articles [19-28] were included in the present systematic review. The study selection process is illustrated in Figure 1.

Study Characteristics

The characteristics table included 10 articles, of which nine were in vitro studies and one was a case report published between 2020 and 2024 [19-28]. In all in vitro studies, extracted human teeth were used, whereas the case report involved the treatment of a maxillary lateral incisor. The included studies assessed different aspects of endodontic retreatment. Two studies focused on post space preparation [19,20], and five studies evaluated fiber post removal [22-24,26,27]. Karim and Faraj [21] assessed the DNS application in the retrieval of separated instruments, and Sajjan et al. [25] investigated the location of the mesiobuccal canal during retreatment.

All studies compared DNS with the FH technique, except Martinho et al. [27], which compared DNS with augmented reality (AR) head-mounted navigation, and Karim and Faraj [21], which used a dental operating microscope for comparison. Outcomes assessed included coronal and angular deviations, angular deflections, volumetric loss of tooth structure, procedural time, and the number of mishaps, providing measures of DNS accuracy and efficiency. Detailed study characteristics are summarized in Table 1.

**Table 1:** Characteristics of the included studies DNS, dynamic navigation system; FH, freehand; NA, not applicable; NR, not reported

S. no.	Author and place	Year and study	Teeth/procedure	Groups and total sample (n)	DNS system	No. and experience of operators	Procedural time (minutes)	Angular deviation (°)	Coronal deviation (mm)	Apical deviation (mm)
1	Martinho et al. [19], Baltimore, Maryland	2024, In vitro	Extracted maxillary molars/post space preparation	1. 3D-DNS 2. FH (n = 27)	X-Guide software	1 trained surgeon	1. DNS – 4.20 ± 0.23 2. FH – 5.14 ± 0.40	1. DNS – 1.70 ± 0.73 2. FH – 3.90 ± 2.11	1. DNS – 0.83 ± 0.40 2. FH – 1.52 ± 1.57	1. DNS – 0.90 ± 0.62 2. FH – 2.40 ± 1.53
2	Shervani et al. [20], India	2024, In vitro	Extracted maxillary central incisors/post space preparation	1. 3D-DNS 2. FH (n = 30)	Navident	Single trained operator	1. DNS – 6.67 ± 1.18 2. FH – 17.23 ± 2.16	1. DNS – 0.85 ± 0.04 2. FH – 2.74 ± 0.75	1. DNS – 0.54 ± 0.13 2. FH – 0.90 ± 0.26	1. DNS – 0.57 ± 0.02 2. FH – 1.05 ± 0.29
3	Loomis [24], South Carolina	2020, In vitro	Maxillary incisors	1. X-Guide 2. FH (n = 15)	X-Guide system	1. Second-year resident 2. Experienced operator	1. DNS – 0.64 ± 0.26 2. FH – 8.73 ± 7.17	1. DNS – 1.14 ± 0.47 2. FH – 3.16 ±	1. DNS – 0.365 ± 0.136 2. FH – 0.621 ± 0.270	Apical non-depth deviation: 1. DNS – 0.298 ± 0.142 2. FH – 0.381 ± 0.315; apical depth deviation: 1. DNS – 0.130 ± 1.106 2. FH – 1.026 ± 1.289
4	Janabi et al. [23], Maryland	2021, In vitro	Maxillary single-rooted teeth / post & core removal	1. 3D-DNS 2. FH (n = 13)	X-Guide software	NR	1. DNS – 4.03 ± 0.43 2. FH – 8.30 ± 4.65	1. DNS – 1.75 ± 0.63 2. FH – 4.49 ± 2.10	1. DNS – 0.91 ± 0.65 2. FH – 1.13 ± 0.84	1. DNS – 1.17 ± 0.64 2. FH – 1.68 ± 0.85
5	Dale [22], South Carolina	2022, In vitro	Maxillary anterior teeth	1. Robot-assisted haptic guidance (n = 16) 2. FH (n = 15)	DICOM	1 experienced, 1 inexperienced	1. Robot-guided – 0.55 ± 0.35 2. FH experienced – 7.43 ± 3.57	NR	NR	NR
6	Banik et al. [26], Jharkhand	2022, IJHS, In vitro	40 single-rooted teeth, root canal treated and restored with Parapost taper & core buildup	1. DNS 2. FH (n = 20)	X-Nav software	NR	1. DNS – 3.525 ± 0.164 2. FH – 1.699 ± 1.324	1. DNS – 86.86 ± 91.71 2. FH – 91.54 ± 86.11	1. DNS – 1.525 ± 0.164 2. FH – 4.699 ± 1.324	1. DNS – 1.525 ± 0.164 2. FH – 4.699 ± 1.324
7	Martinho et al. [27], Baltimore, Maryland	2024, JOE, In vitro	Extracted maxillary molars	1. AR HMD-DNS 2. DNS (n = 25)	X-Guide software	Both experienced operators	1. DNS – 4.2 2. FH – 4.5	1. DNS – 1.3 2. FH – 2.3	1. DNS – 1.3 2. FH – 1.8	1. DNS – 1.2 2. FH – 2.2
8	Sajjan et al. [25], Andhra Pradesh	2024, IJHS, In vitro	20 root canal–treated human mandibular molars	Location of mesiobuccal canal: 1. FH 2. DNS (n = 10)	Navident	NR	1. DNS – 53.60 2. FH – 29.00	NR	NR	NR
9	Karim and Faraj [21], Iraq	2023, JOE, In vitro	30 extracted maxillary first bicuspids with 60 separated roots	1. DOM 2. DNS (n = 15)	Navident	NR	1. DNS – 23 ± 6.67 2. FH – 12.1 ± 7.25	1. DNS – 1.75 ± 0.63 2. FH – 4.49 ± 2.10	1. DNS – 0.91 ± 0.65 2. FH – 0.13 ± 0.84	1. DNS – 1.17 ± 0.64 2. FH – 1.68 ± 0.85
10	Bardales-Alcocer et al. [28], Mexico	2021, JOE, Case report	Previously treated left maxillary lateral incisor with a fiber post and a zirconia crown	NA	Navident	NA	NA	NA	NA	NA

Quality Assessment

**Figure 2:**
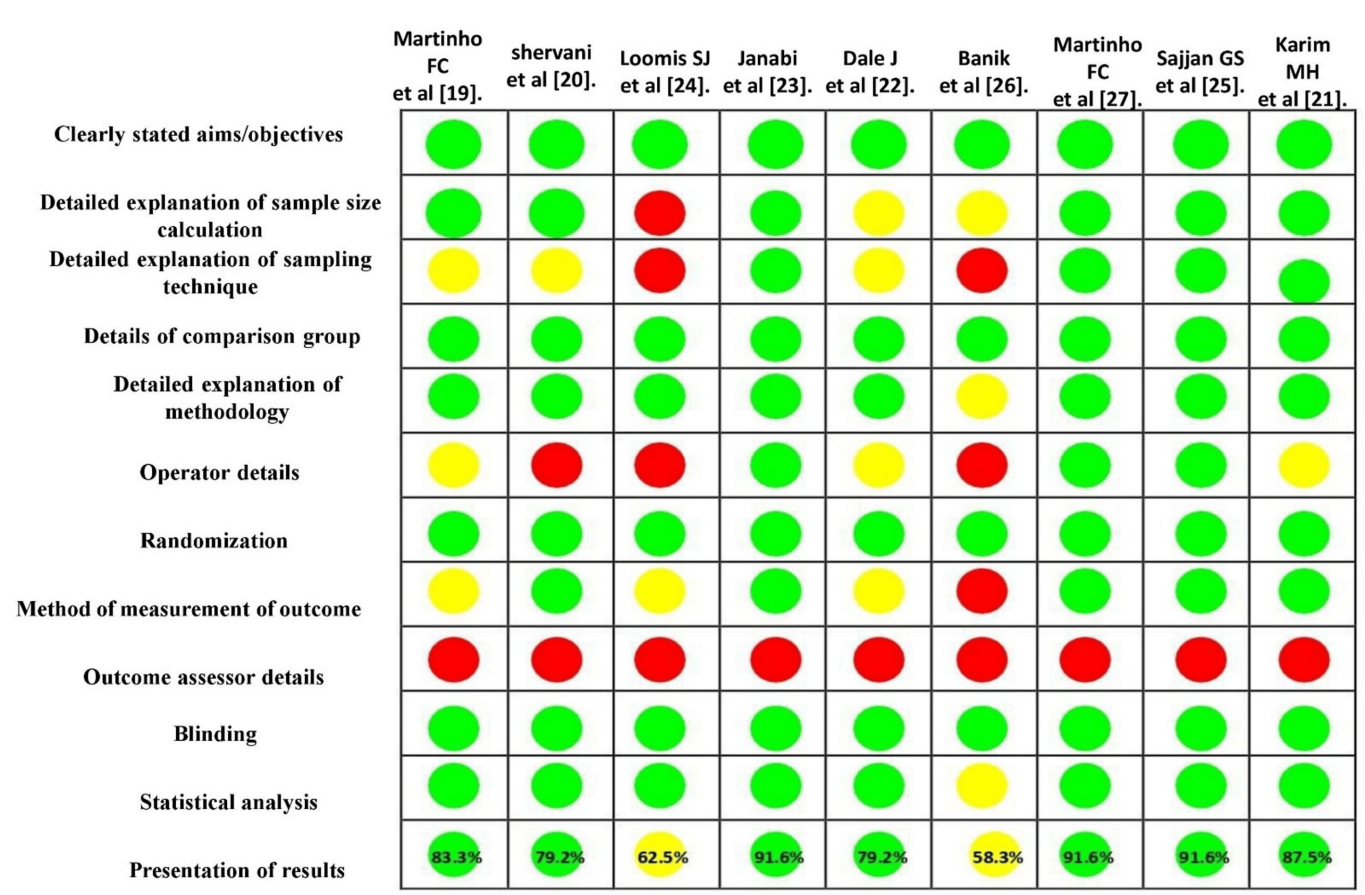
Detailed quality assessment using the QUIN tool Green indicates a score of adequately specified (2), yellow indicates not adequately specified (1), red indicates not specified (0), and NA represents not applicable. Studies were classified as low risk (>70%, green), medium risk (50-70%, yellow), or high risk (<50%, red). QUIN tool, Quality Assessment Tool for In Vitro Studies

The risk of bias for the nine included in vitro studies was assessed using the QUIN tool. Among these studies, seven were classified as low risk of bias, while two studies, Loomis [24] and Banik et al. [26], were categorized as medium risk of bias, with scores of 62.5% and 54.1%, respectively. No study was classified as high risk. The detailed quality assessment is presented in Figure 2.

Quantitative Assessment: Meta-Analysis

**Figure 3:**
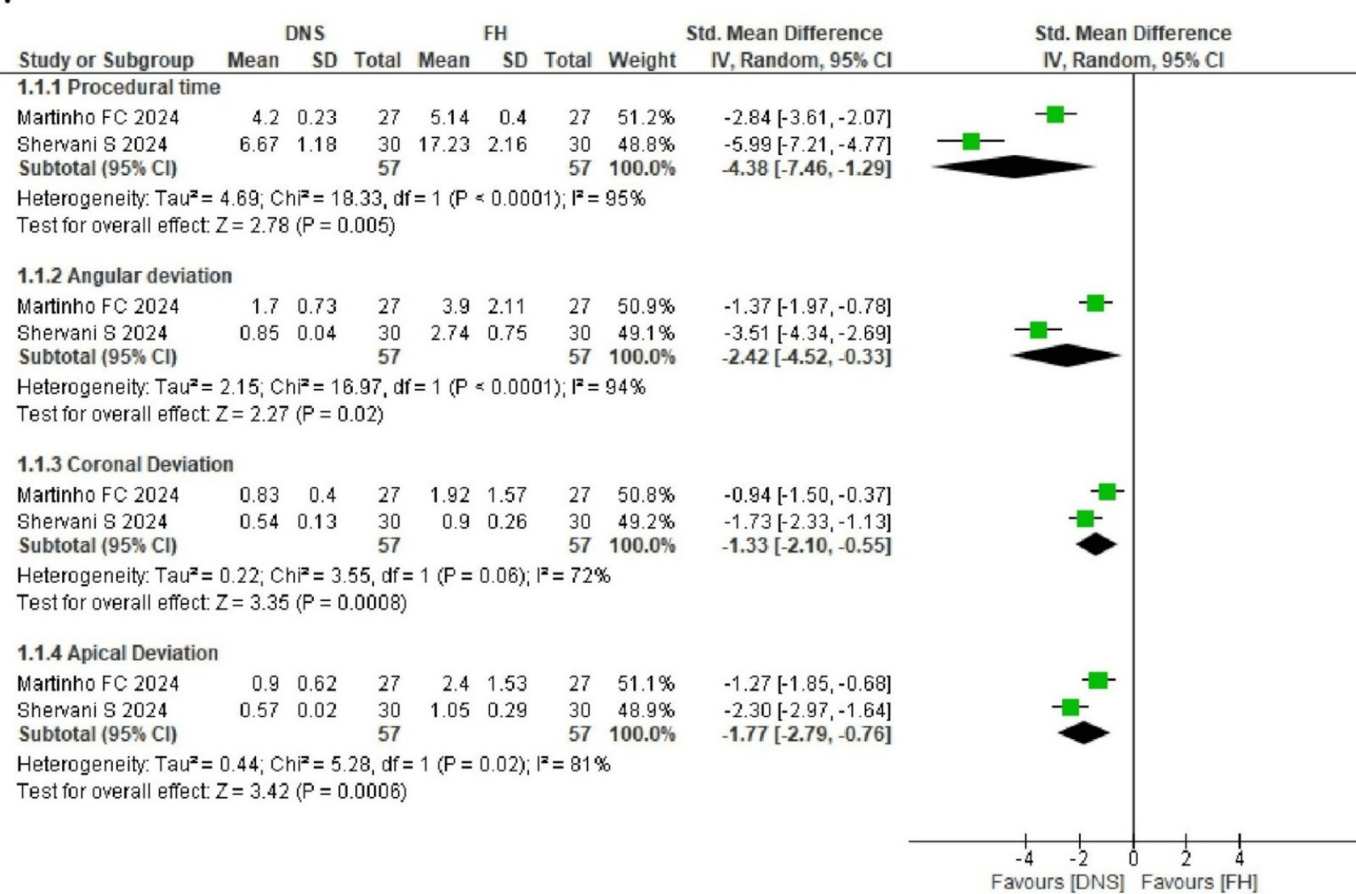
Forest plot for post space preparation The studies included were Martinho et al. [19] and Shervani et al. [20].

Two forest plots using the random effects model were generated with Review Manager (RevMan) version 5.4 (Cochrane Collaboration, Oxford, UK) for post space preparation and post removal. Different outcomes, including procedural time, angular deviation, coronal deviation, and apical deviation, were analyzed. Meta-analysis was performed using mean and standard deviation values with 95% confidence intervals. Heterogeneity was assessed using I² values, and either fixed or random effects models were applied based on these results. A total of five studies were included in the meta-analysis: two for post space preparation [19,20] and three for post removal [22-24]. Figure 3 shows the forest plot for post space preparation.

Post Space Preparation

Procedural time: Analysis using the standard mean difference and random effects model showed high heterogeneity (I² = 95%, p < 0.0001, 95% CI). The DNS required 4.2 and 6.67 minutes to prepare post space, whereas the FH technique took 5.14 to 17.53 minutes.

Angular deviation: Angular deviation ranged from 0.85 to 1.7° for DNS and 2.74 to 3.9° for FH. The standard mean difference and random effects model were applied due to high heterogeneity (I² = 94%, p < 0.0001, 95% CI).

Coronal deviation: Coronal deviation ranged from 0.54 to 0.83 mm for DNS and 0.9 to 1.92 mm for FH. The pooled estimate showed I² = 72% and p = 0.06, indicating high heterogeneity; therefore, the random effects model was applied.

Apical deviation: Apical deviation ranged from 0.57 to 0.9 mm for DNS and 1.05 to 2.04 mm for FH. High heterogeneity (I² = 81%, p = 0.02, 95% CI) necessitated the use of the random effects model.

**Figure 4:**
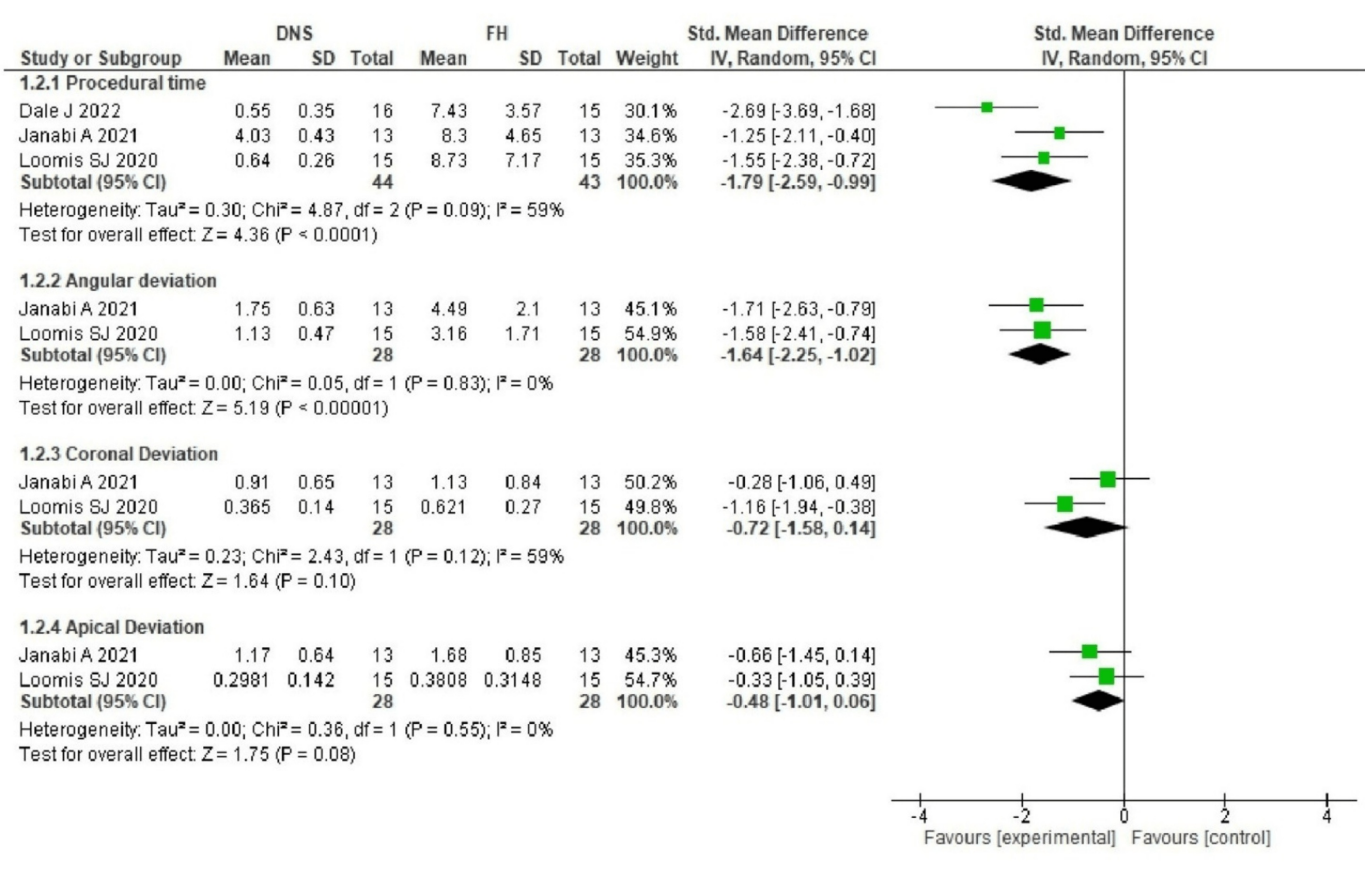
Forest plot for post removal The studies included were Dale [22], Janabi et al. [23], and Loomis [24].

Figure 4 shows the forest plot for post removal.

Post Removal

Procedural time: The time required for post removal ranged from 0.55 to 4.03 minutes for DNS, whereas the FH technique took 7.43 to 8.73 minutes. Due to high heterogeneity (I² = 59%, p < 0.09, 95% CI), the standard mean difference was analyzed using a random effects model.

Angular deviation: DNS showed lower angular deviation, ranging from 1.13 to 1.75°, compared to 3.16 to 4.49° for the FH technique. Zero heterogeneity (I² = 0%, 95% CI) allowed the use of a fixed effects model.

Coronal deviation: Coronal deviation for DNS ranged from 0.365 to 0.91 mm, while for the FH technique, it ranged from 0.621 to 1.13 mm. High heterogeneity (I² = 59%, p = 0.12, 95% CI) required analysis using a random effects model.

Apical deviation: Apical deviation was lower for DNS, ranging from 0.29 to 1.17 mm, compared to 0.38 to 1.68 mm for the FH technique. Zero heterogeneity (I² = 0%) allowed the use of a fixed effects model.

Discussion

The DNS is a cutting-edge innovation that enhances precision and efficiency in complex endodontic procedures [29]. Typically, it comprises a mobile workstation, a display screen, an interactive software interface for treatment planning and navigation, and a 3D spatial tracking system to monitor positioning [30]. There are two generations of DNS [31]. The first-generation workflow involves four steps: stent, scan, plan, and place. Commercially available systems include NAVIDENT, X-Guide, Image Yomi, Inliant, and X-NAV. Drawbacks of first-generation DNS include high cost, immature design, and limited access to CT scanners. With improvements in computing and optical tracking technology, second-generation systems emerged, introducing trace registration. This feature identifies anatomical and artificial landmarks visible in scans, such as teeth, implants, abutments, bone ridges, and fixation screws, with a simplified workflow called TAP (Trace and Place), exemplified by NAVIDENT 2.0. In the studies included in this systematic review, the most commonly used DNS were Navident and X-Guide.

DNS serves as a guiding tool in procedures such as pulp canal obliteration, endodontic retreatment, and microsurgery. Endodontic retreatment primarily involves the removal of existing obturation materials, retrieval of separated instruments, correction of iatrogenic errors, and post removal. While several systematic reviews have examined the use of DNS in microsurgical and nonsurgical endodontic treatment, none have specifically focused on endodontic retreatment. This systematic review aimed to evaluate the effectiveness of DNS in retreatment procedures compared to conventional FH techniques.

A total of 10 studies met the eligibility criteria for inclusion in this review, of which nine were in vitro studies and one was a case report. The studies assessed various applications: post space preparation in two studies [19,20], post removal in five studies [22-24,26,27], removal of separated instruments in one study [21], and location of the second mesiobuccal canal in maxillary molars in one study [25].

Of these 10 studies, five were included in the meta-analysis. Excluded studies were Bardales-Alcocer et al. [28], a case report; Sajjan et al. [25] and Karim and Faraj [21], which assessed different DNS applications; Martinho et al. [27], which evaluated AR head-mounted devices without an FH comparison; and Banik et al. [26], excluded due to discrepancies in outcome data and low study quality.

The meta-analysis showed that DNS required 0.55 to 4.03 minutes for post removal and 4.2 to 6.67 minutes for post space preparation, significantly less than FH techniques, which required 7.43 to 8.73 minutes and 5.14 to 17.23 minutes, respectively. Angular deviation with DNS ranged from 0.85 to 1.7° for post space preparation and 1.13 to 1.75° for post removal, whereas FH showed greater deviation (2.74-3.9° and 3.16-4.49°, respectively). Coronal deviation for DNS ranged from 0.54 to 0.83 mm in post space preparation and 0.36 to 0.91 mm in post removal, compared to 0.9 to 1.92 mm and 0.621 to 1.13 mm with FH. Apical deviation with DNS ranged from 0.57 to 0.9 mm in post space preparation and 0.29 to 1.17 mm in post removal, whereas FH showed 1.05 to 2.04 mm and 0.38 to 1.68 mm, respectively.

The case report by Bardales-Alcocer et al. [28] demonstrated the use of DNS in removing a fiber post from a maxillary lateral incisor with high accuracy and minimal tooth structure loss. Sajjan et al. [25] reported that locating the second mesiobuccal canal in maxillary molars during retreatment required significantly less time and resulted in minimal tissue loss with DNS compared to FH. Karim and Faraj [21] found that a 3D microscope outperformed DNS in the retrieval of broken rotary NiTi files in terms of success rate, time, and canal aberration.

Operator experience and training with DNS also influenced procedural time and errors. Five studies reported correlations between operator experience and time. Martinho et al. [19] noted that minimal deviations still occurred due to human error despite the computer-assisted nature of DNS. Shervani et al. [20], Loomis [24], Martinho et al. [27], Karim and Faraj [21], and Bardales-Alcocer et al. [28] reported no impact of operator experience on DNS performance, acknowledging a steep learning curve. Dale [22] observed a significant difference in procedural time between experienced and inexperienced operators, attributable to pre-setup duration. Banik et al. [26] reported that combining operator experience with DNS reduced procedural time compared to FH. Although most included studies were in vitro, the overall risk of bias was limited to medium.

Limitations

The limitations of this systematic review and meta-analysis include the small number of available studies. Only in vitro studies and case reports were included, as no randomized clinical trials have been reported in the literature. Additionally, some of the measured outcomes exhibited a high degree of heterogeneity.

Future Perspectives

The success of DNS is often influenced by the operator’s experience, training, and skill, highlighting the importance of adequate training. Since no clinical trials currently exist, there is a need for high-quality evidence from randomized clinical trials to determine the clinical outcomes of DNS. Future studies should compare multiple operators and different DNS systems, including static guidance, to provide a comprehensive evaluation.

## Conclusions

Within the limitations of this systematic review and meta-analysis, DNS outperformed the FH technique across all measured outcomes, including reduced procedural time, lower angular deviation, and minimal coronal and apical deviation and mishaps during post space preparation and post removal procedures. These findings demonstrate that DNS offers high precision and efficiency, making it particularly valuable for complex endodontic procedures that require meticulous accuracy. When operators are appropriately trained, the use of DNS reduces procedural errors and the risk of compromising vital tooth structures, all within a shorter timeframe compared to the FH technique. Nonetheless, robust clinical trials are necessary to validate the results observed in existing in vitro studies.

## Appendices

**Table 2:** Detailed database search DNS, dynamic navigation system

Database	Search strategy
PubMed	(Retreatment) OR (rootcanal retreatment)) OR (root canal retreatment)) OR (endodontic retreatment)) OR (endodontic post removal OR endodontic postremoval)) OR (endodontic file retrieval OR endodontic file removal OR separated file removal)) AND ((((((dynamic navigation) OR (computer aided technology)) OR (computer aided navigation)) OR (computer assisted treatment)) OR (image-guided treatment)) OR (real-time tracking))
Scopus	("dynamic navigation" OR "computer-aided navigation" OR "guided endodontics" OR DNS OR "real-time navigation") AND ("endodontic retreatment" OR "root canal retreatment" OR "non-surgical retreatment"))
Web of science	(“dynamic navigation” OR “dynamic navigation system” OR “computer-aided navigation” OR “real-time navigation” OR “guided endodontics” OR “computer-assisted treatment” OR “image-guided treatment” OR “navigation system”) AND (“endodontic retreatment” OR “root canal retreatment” OR “non-surgical retreatment” OR “surgical endodontics” OR “retrograde root canal treatment” OR “calcified canal”)
Cochrane	("dynamic navigation system" OR "dynamic navigation" OR "computer-aided navigation" OR "guided endodontics" OR DNS OR "real-time guided endodontics" OR "computer-assisted treatment" OR "navigation system") AND("endodontic retreatment" OR "root canal retreatment" OR "non-surgical retreatment" OR "dental post removal" OR "fiber post removal" OR "broken instrument retrieval" OR "calcified canal")
